# Association of four genetic polymorphisms in the vascular endothelial growth factor-A gene and development of ovarian cancer: a meta-analysis

**DOI:** 10.18632/oncotarget.20379

**Published:** 2017-08-21

**Authors:** Chao-Huan Xu, Zhong-Hui He, Hong Xu

**Affiliations:** ^1^ Department of Gynaecology, First Affiliated Hospital of Guangxi Medical University, Nanning 530021, China

**Keywords:** vascular endothelial growth factor, polymorphism, ovarian cancer, meta-analysis

## Abstract

This study meta-analyzed the literature on possible association of four polymorphisms (+936C/T, −460C/T, −2578C/A and −1154G/A) in the vascular endothelial growth factor (VEGF)-A gene with risk of ovarian cancer. Meta-analysis of 7 case-control studies involving +936C/T, 4 studies involving −460C/T, 4 studies involving −2578C/A and 2 studies involving −1154G/A showed significant association between −460C/T and ovarian cancer risk. This risk was observed in the total population (allelic model, OR 1.80, 95% CI 1.26–2.59, *P* = 0.001; recessive model, OR 1.84, 95% CI 1.13–2.98, *P* = 0.01; dominant model, OR 0.51, 95% CI 0.39–0.67, *P* < 0.001; homozygous model, OR 2.48, 95% CI 1.72–3.56, *P* < 0.001; heterozygous model, OR 1.67, 95% CI 1.26–2.21, *P* < 0.001) and in the subgroup of Asian study participants. The CA genotype at −2578C/A was a risk factor in the total population, while the CT genotype at +936C/T was a protective factor in Caucasians. None of the five genetic models suggested a significant association between −1154G/A and ovarian cancer risk in the entire study population, or between +936C/T and risk in Asian or Chinese participants. These findings should be verified in large, well-designed studies.

## INTRODUCTION

Ovarian cancer is a major cause of cancer-related death in females worldwide [1, 2]. Although treatment can significantly improve quality of life, the 5-year survival rate for patients with advanced ovarian cancer remains below 30%, mainly due to high rates of recurrence and metastasis [3, 4]. Development of ovarian cancer has been linked to numerous environmental and lifestyle factors, including age, early menarche, late menopause, non-child-bearing, high-fat diet, exposure to talcum powder and asbestos, and long-term hormone supplementation [5, 6]. Ovarian cancer has also been linked to several genetic polymorphisms [7–9].

Angiogenesis, which refers to the formation of new capillary blood vessels from preexisting ones, is an important factor in the development and spread of cancer, including ovarian cancer [10–12]. A key mediator of angiogenesis is vascular endothelial growth factors (VEGFs) [13], which are expressed at higher levels in malignant ovarian tumor tissues than in benign tumor tissues or tissue of low malignant potential [14–16]. This implicates VEGFs in the pathological angiogenesis of ovarian cancer. Indeed, prognosis and overall survival of ovarian cancer patients correlate with serum and/or tumor levels of VEGFs [17–21]. These findings suggest that genetic factors affecting VEGF expression or activity may influence ovarian cancer development and progression.

The founding member of the VEGF family, VEGF-A, is encoded by a gene on chromosome 6p12 that comprises a 14-kb coding region of eight exons and exhibits alternate splicing to form a family of proteins. [22]. Several single-nucleotide polymorphisms (SNPs) in this gene correlate with VEGF expression [23–25]. Numerous case-control studies [26–32] have investigated whether polymorphisms in the VEGF-A gene at positions +936C/T (rs3025039), −460C/T (rs833061), −2578C/A (rs699947) or −1154G/A (rs1570360) influence ovarian cancer risk. Results have been inconclusive and contradictory, prompting us to perform this comprehensive meta-analysis of all available evidence on these potential associations. To the best of our knowledge, this is the first meta-analysis concerning all four of these previously analyzed polymorphisms and ovarian cancer risk.

## RESULTS

### Description of studies

**Figure 1:**
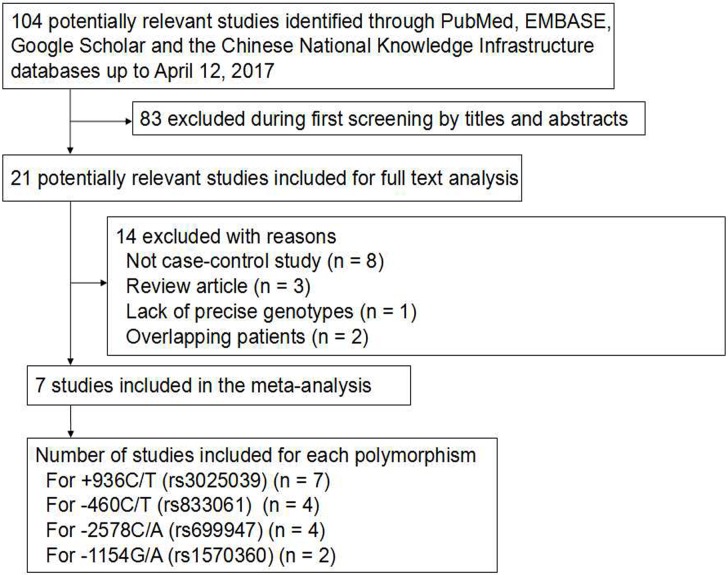
Flowchart of study selection

A total of 104 potentially relevant publications published in English or Chinese up to April 12, 2017 were systematically identified in PubMed, EMBASE, Google Scholar and Chinese National Knowledge Infrastructure databases (Figure 1). We excluded 83 studies based on review of the titles and abstracts, because they did not analyze the target polymorphisms in the VEGF-A gene or because they did not examine ovarian cancer risk. We excluded another 8 studies because they were not case-control studies, 3 studies because they were review articles and 1 study because it did not report precise genotypes. Another 2 studies were excluded because they analyzed overlapping patient populations. In the end, 7 studies were included in the final meta-analysis [26–32] (Table 1).

**Table 1:** Characteristics of studies in the meta-analysis

First author	Year	Ethnicity	Country	Genotyping method	Type of controls	P for HWE	Cases/Controls	No. of cases			Allele frequencies of cases, n, (%)		No. of controls			Allele frequencies of controls, n, (%)	
+936C/T (rs3025039)								CC	CT	TT	C	T	CC	CT	TT	C	T
Konac [26]	2007	Caucasian	Turkey	PCR-RFLP	PB	0.156	47/106	1	13	33	15 (16.0)	79 (84.0)	1	34	71	36 (17.0)	176 (83.0)
Jakubowska [27]	2008	Caucasian	Poland	PCR-RFLP	HB	0.863	145/280	108	33	4	249 (85.9)	41 (14.1)	196	77	7	469 (83.8)	91 (16.2)
Jia [28]	2009	Asian	China	PCR-RFLP	HB	0.729	256/329	174	77	5	425 (83.0)	87 (17.0)	229	92	8	550 (83.6)	108 (16.4)
Li [29]	2010	Asian	China	PCR-RFLP	HB	0.443	303/303	211	86	6	508 (83.8)	98 (16.2)	201	94	8	496 (81.8)	110 (18.2)
RinckJunior [30]	2015	Caucasian	Brazil	PCR-RFLP	HB	0.343	131/137	111	16	4	238 (90.8)	24 (91.6)	103	33	1	239 (87.2)	35 (12.8)
Janardhan [31]	2015	Asian	India	PCR-RFLP	PB	0.625	300/320	232	64	4	528 (88.0)	72 (12.0)	303	17	0	623 (97.3)	17 (2.7)
Zhang[32]	2016	Asian	China	PCR-RFLP	HB	0.616	163/276	109	46	8	264 (81.0)	62 (19.0)	192	75	9	459 (83.1)	93 (16.9)
−460C/T (rs833061)								CC	CT	TT	C	T	CC	CT	TT	C	T
Konac [26]	2007	Caucasian	Turkey	PCR-RFLP	PB	0.156	47/106	5	21	21	15 (16.0)	79 (84.0)	13	58	35	84 (39.6)	128 (60.4)
Li [29]	2010	Asian	China	PCR-RFLP	HB	0.262	303/303	12	93	198	117 (19.3)	489 (80.7)	17	95	191	129 (21.3)	477 (78.7)
Janardhan [31]	2015	Asian	India	PCR-RFLP	PB	0.945	300/320	96	152	52	344 (57.3)	256 (42.7)	167	128	25	462 (72.2)	178 (27.8)
Zhang[32]	2016	Asian	China	PCR-RFLP	HB	0.002	163/176	13	63	87	89 (27.3)	237 (72.7)	19	104	53	142 (40.3)	210 (59.7)
−2578C/A (rs699947)								CC	CA	AA	C	A	CC	CA	AA	C	A
Jia [28]	2009	Asian	China	PCR-RFLP	HB	0.155	256/329	140	99	17	379 (74.0)	133 (26.0)	191	113	25	495 (75.2)	163 (24.8)
Li [29]	2010	Asian	China	PCR-RFLP	HB	0.807	303/303	166	117	20	449 (74.1)	157 (25.9)	183	104	16	470 (77.6)	136 (22.4)
Janardhan [31]	2015	Asian	India	PCR-RFLP	PB	0.886	300/320	116	142	42	374 (62.3)	226 (37.7)	117	154	49	388 (60.6)	252 (39.4)
Zhang[32]	2016	Asian	China	PCR-RFLP	HB	0.257	163/276	90	58	15	238 (73.0)	88 (27.0)	200	67	9	467 (84.6)	85 (15.4)
−1154G/A (rs1570360)								GG	GA	AA	G	A	GG	GA	AA	G	A
Li [29]	2010	Asian	China	PCR-RFLP	HB	0.952	302/303	244	54	4	542 (89.7)	62 (10.3)	217	79	7	513 (84.7)	93 (15.3)
Janardhan [31]	2015	Asian	India	PCR-RFLP	PB	0.425	300/320	166	113	21	445 (74.2)	155 (25.8)	239	77	4	555 (86.7)	85 (13.3)

PCR, polymerase chain reaction; RFLP, restriction fragment length polymorphism; HWE, Hardy-Weinberg equilibrium; PB, population-based; HB, hospital-based.

All 7 studies evaluated the association between the +936C/T polymorphism and ovarian cancer risk (1,345 cases and 1,671 controls). Four studies [26, 29, 31, 32] evaluated the association between the −460C/T polymorphism and ovarian cancer risk (813 cases and 905 controls); 4 studies [28, 29, 31, 32], the association between the −2578C/A polymorphism and ovarian cancer risk (1,022 cases and 1,228 controls); and 2 studies [29, 31], the association between the −2578C/A polymorphism and ovarian cancer risk (602 cases and 623 controls). The distribution of genotypes in controls was consistent with Hardy-Weinberg equilibrium (HWE, *P* > 0.05) in all but one study [32] involving the −460C/T polymorphism.

All studies in the meta-analysis received a score of at least 6 on the Newcastle–Ottawa Scale [34], indicating that they were all of good quality. The mean score for all included studies was 7 (Table 2).

**Table 2:** Methodological quality of case-control studies in our meta-analyses, based on the Newcastle–Ottawa Scale

Selection (score)					Comparability(score)	Exposure (score)			Total score^b^
Study	Adequate definition of patient cases	Representativeness of patients/cases	Selection of controls	Definition of controls	Control for importantfactor or additional factor	Ascertainment of exposure (blinding)	Same method of ascertainment for participants	Non-response rate^a^	
Konac [26]	1	1	1	1	2	0	1	1	8
Jakubowska [27]	1	1	0	1	2	0	1	1	7
Jia [28]	1	1	0	1	1	0	1	1	6
Li [29]	1	1	0	1	1	0	1	1	6
Rinck-Junior [30]	1	1	0	1	1	0	1	1	6
Janardhan [31]	1	1	1	1	2	0	1	1	8
Zhang [32]	1	1	1	1	2	0	1	1	8

^a^ One point was awarded when there was no significant difference in the response rate between groups, based on the chi-squared test (*P* > 0.05).

^b^ Calculated by adding up the points awarded for each item.

### Meta-analysis of studies on the +936C/T (rs3025039) polymorphism

**Figure 2:**
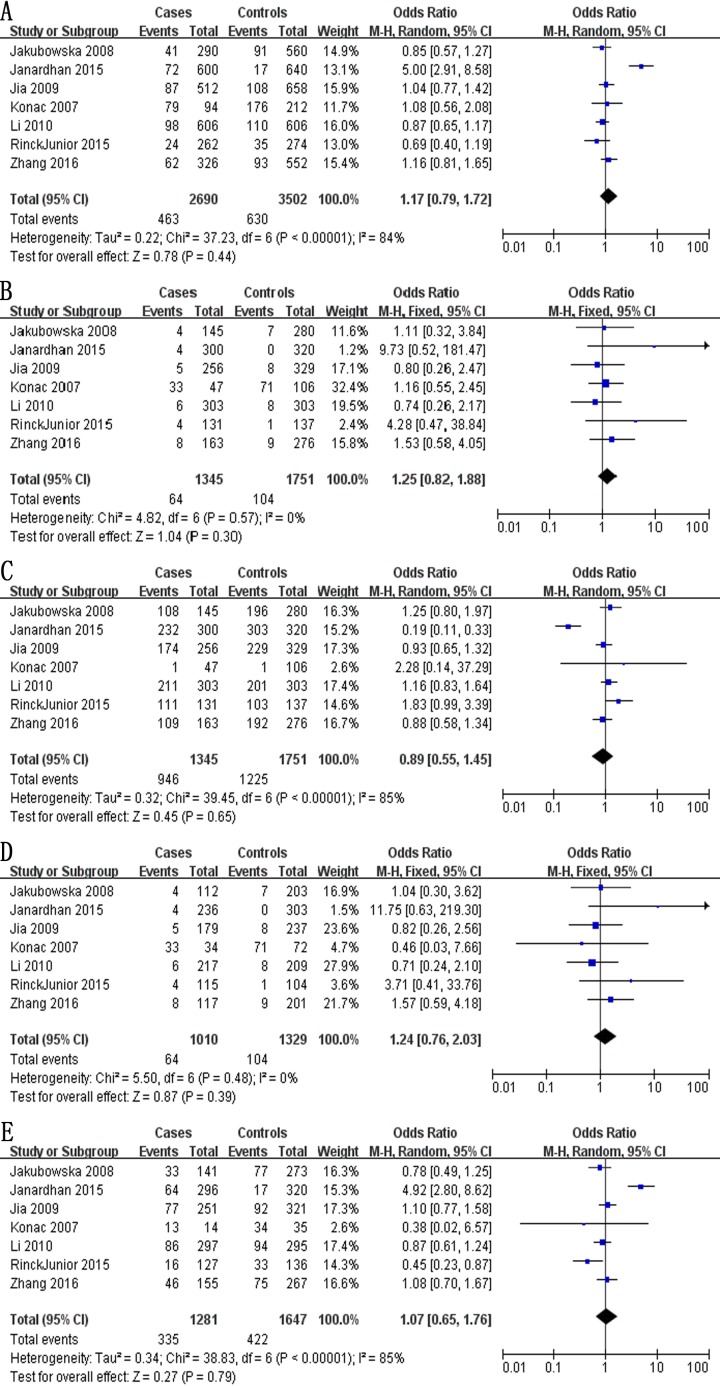
Forest plot describing the association between the +936C/T polymorphism (rs3025039) and risk of ovarian cancer across all study participants according to five genetic models (**A**) allelic (T-allele vs. C-allele), (**B**) recessive (TT vs. CT + CC), (**C**) dominant (CC vs. CT + TT), (**D**) homozygous (TT vs. CC) and (**E**) heterozygous (CT vs. CC).

Meta-analysis of a possible association between +936C/T polymorphism and ovarian cancer risk is summarized in Table 3. Based on the total study population involving 1,345 cases and 1,751 controls, none of the five genetic models indicated a significant association: allelic model, OR 1.17, 95% CI 0.79–1.72, *P* = 0.44 (Figure 2A); recessive model, OR 1.25, 95% CI 0.82–1.88, *P* = 0.30 (Figure 2B); dominant model, OR 0.89, 95% CI 0.55–1.45, *P* = 0.65 (Figure 2C); homozygous model, OR 1.24, 95% CI 0.76–2.03, *P* = 0.39 (Figure 2D); and heterozygous model, OR 1.07, 95% CI 0.65–1.76, *P* = 0.79 (Figure 2E).

**Table 3:** Overall meta-analysis of the association between the +936C/T (rs3025039) and risk of ovarian cancer

Genotype comparison and genetic model	OR [95 % CI]	Z (*P* value)	Heterogeneity of study design			Analysis model
			χ^2^	df (*P* value)	I^2^(%)	
**+936C/T (rs3025039) in total population from 7 case control studies (1,345 cases and 1,751 controls)**						
Allelic (T-allele vs. C-allele)	1.17 [0.79, 1.72]	0.78 (0.44)	37.23	6 (< 0.001)	84	Random
Recessive (TT vs. CT + CC)	1.25 [0.82, 1.88]	1.04 (0.30)	4.82	6 (0.57)	0	Fixed
Dominant (CC vs. CT + TT)	0.89 [0.55, 1.45]	0.45(0.65)	39.45	6 (< 0.001)	85	Random
Homozygous (TT vs. CC)	1.24 [0.76, 2.03]	0.87 (0.39)	5.50	6 (0.48)	0	Fixed
Heterozygous (CT vs. CC)	1.07 [0.65, 1.76]	0.27 (0.79)	38.83	6 (< 0.001)	85	Random
**+936C/T (rs3025039) in Asian population from 4 case-control studies (1,022 cases and 1,228 controls)**						
Allelic (T-allele vs. C-allele)	1.47 [0.81, 2.67]	1.25 (0.21)	33.47	3 (< 0.001)	91	Random
Recessive (TT vs. CT + CC)	1.19 [0.68, 2.11]	0.61 (0.54)	3.46	3 (0.33)	13	Fixed
Dominant (CC vs. CT + TT)	0.67 [0.35, 1.29]	1.19 (0.23)	30.92	3 (< 0.001)	90	Random
Homozygous (TT vs. CC)	1.22 [0.69, 2.16]	0.68 (0.50)	3.96	3 (0.27)	24	Fixed
Heterozygous (CT vs. CC)	1.46 [0.77, 2.75]	1.16 (0.24)	27.99	3 (< 0.001)	89	Random
**+936C/T (rs3025039) in Caucasian population from 3 case-control studies (323 cases and 523 controls)**						
Allelic (T-allele vs. C-allele)	0.84 [0.63, 1.12]	1.19 (0.23)	1.05	2 (0.59)	0	Fixed
Recessive (TT vs. CT + CC)	1.31 [0.71, 2.39]	0.87 (0.38)	1.28	2 (0.53)	0	Fixed
Dominant (CC vs. CT + TT)	1.44 [1.00, 2.07]	1.98 (0.05)	1.07	2 (0.59)	0	Fixed
Homozygous (TT vs. CC)	1.31 [0.50, 3.44]	0.55 (0.58)	1.51	2 (0.47)	0	Fixed
Heterozygous (CT vs. CC)	0.64 [0.44, 0.93]	2.33 (0.02)	1.90	2 (0.39)	0	Fixed
**+936C/T (rs3025039) in Chinese population from 3 case-control studies (722 cases and 908 controls)**						
Allelic (T-allele vs. C-allele)	1.00 [0.83, 1.20]	0.01 (0.99)	1.57	2 (0.46)	0	Fixed
Recessive (TT vs. CT + CC)	1.00 [0.55, 1.83]	0.00 (1.00)	1.18	2 (0.55)	0	Fixed
Dominant (CC vs. CT + TT)	1.00 [0.81, 1.23]	0.01 (0.99)	1.28	2 (0.53)	0	Fixed
Homozygous (TT vs. CC)	1.00 [0.55, 1.84]	0.01 (1.00)	1.29	2 (0.52)	0	Fixed
Heterozygous (CT vs. CC)	1.00 [0.81, 1.24]	0.01 (0.99)	0.99	2 (0.61)	0	Fixed

OR, odds ratio; 95% CI, 95% confidence interval.

We also meta-analyzed data for ethnic subgroups. Meta-analysis of 4 studies [28, 29, 31, 32] involving 1,022 Asian cases and 1,228 Asian controls showed no evidence of a significant association between the +936C/T polymorphism and ovarian risk risk for any of the five genetic models (Table 3): allelic, OR 1.47, 95% CI 0.81–2.67, *P* = 0.21; recessive model, OR 1.19, 95% CI 0.68–2.11, *P* = 0.54; dominant, OR 0.67, 95% CI 0.35–1.29, *P* = 0.23; homozygous, OR 1.22, 95% CI 0.69–2.16, *P* = 0.50; and heterozygous, OR 1.46, 95% CI 0.77–2.75, *P* = 0.24. Similarly, no evidence of an association was identified in meta-analysis of 3 studies [26, 27, 30] involving 323 Caucasian cases and 523 Caucasian controls in four genetic models: allelic, OR 0.84, 95% CI 0.63–1.12, *P* = 0.23; recessive, OR 1.31, 95% CI 0.71–2.39, *P* = 0.38; dominant, OR 1.44, 95% CI 1.00–2.07, *P* = 0.05; and homozygous, OR 1.31, 95% CI 0.50–3.44, *P* = 0.58. In contrast, the CT genotype at +936C/T was found to be a protective factor in the heterozygous model (OR 0.64, 95% CI 0.44–0.93, *P* = 0.02; Table 3). Lastly, meta-analysis of 3 studies [28, 29, 32] involving 722 Chinese cases and 908 Chinese controls showed no evidence of a significant association between the +936C/T polymorphism and ovarian risk for any of the five genetic models (Table 3): allelic, OR 1.00, 95% CI 0.83–1.20, *P* = 099; recessive, OR 1.00, 95% CI 0.55–1.83, *P* = 1.00; dominant, OR 1.00, 95% CI 0.81–1.23, *P* = 0.99; homozygous, OR 1.00, 95% CI 0.55–1.84, *P* = 1.00; and heterozygous, OR 1.00, 95% CI 0.81–1.24, *P* = 0.99.

### Meta-analysis of studies on the −460C/T (rs833061) polymorphism

**Figure 3:**
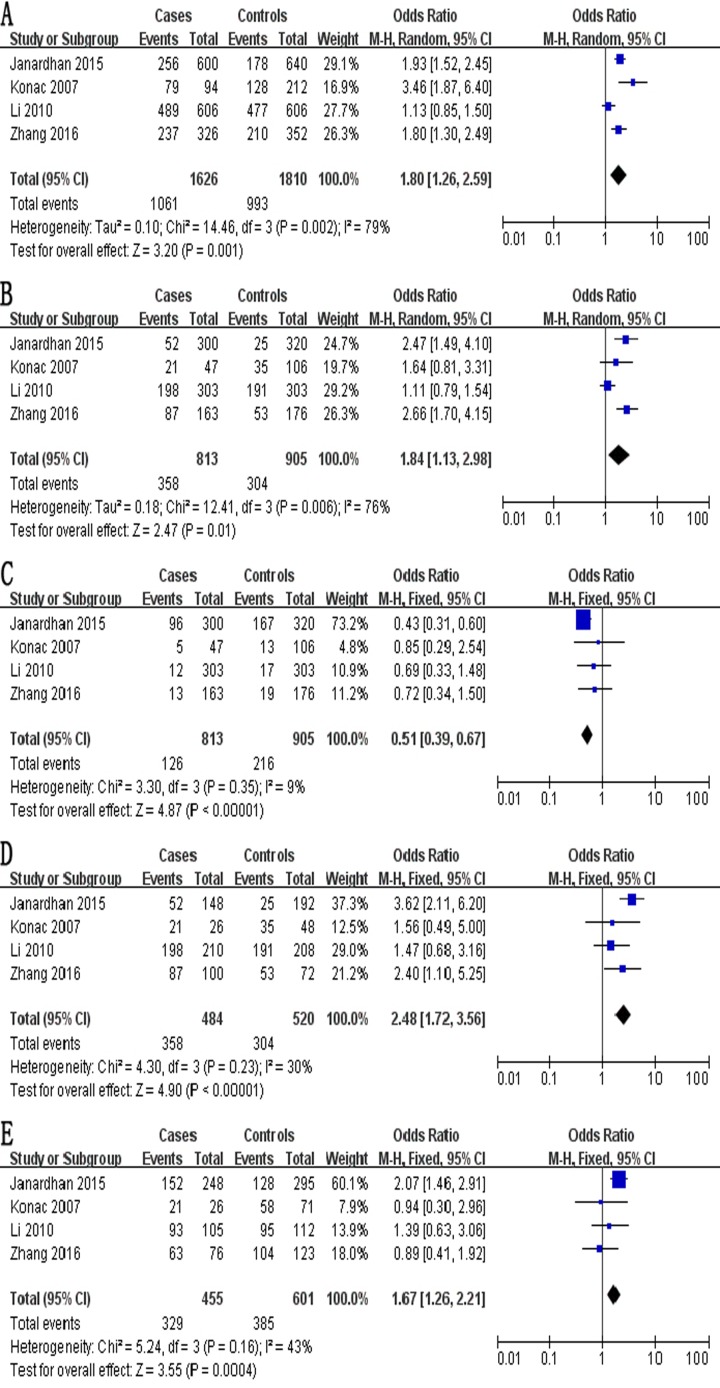
Forest plot describing the association between the −460C/T polymorphism (rs833061) and risk of ovarian cancer across all study participants according to five genetic models (**A**) allelic (T-allele vs. C-allele), (**B**) recessive (TT vs. CT + CC), (**C**) dominant (CC vs. CT + TT), (**D**) homozygous (TT vs. CC) and (**E**) heterozygous (CT vs. CC).

The meta-analysis of a possible association between the −460C/T polymorphism and ovarian risk is summarized in Table 4. Based on the total study population involving 813 cases and 905 controls, a significant association between the −460C/T polymorphism and ovarian risk was demonstrated across the total population according to five genetic models: allelic, OR 1.80, 95% CI 1.26–2.59, *P* = 0.001 (Figure 3A); recessive, OR 1.84, 95% CI 1.13–2.98, *P* = 0.01 (Figure 3B); dominant, OR 0.51, 95% CI 0.39–0.67, *P* < 0.001 (Figure 3C); homozygous, OR 2.48, 95% CI 1.72–3.56, *P* < 0.001 (Figure 3D); and heterozygous, OR 1.67, 95% CI 1.26–2.21, *P* < 0.001 (Figure 3E).

**Table 4:** Overall meta-analysis of the association between the −460C/T (rs833061) and risk of ovarian cancer

Genotype comparison and genetic model	OR [95 % CI]	Z (*P* value)	Heterogeneity of study design			Analysis model
			χ^2^	df (*P* value)	I^2^(%)	
**−460C/T (rs833061) in total population from 4 case-control studies (813 cases and 905 controls)**						
Allelic (T-allele vs. C-allele)	1.80 [1.26, 2.59]	3.20 (0.001)	14.46	3 (0.002)	79	Random
Recessive (TT vs. CT + CC)	1.84 [1.13, 2.98]	2.47 (0.01)	12.41	3 (0.006)	76	Random
Dominant (CC vs. CT + TT)	0.51 [0.39, 0.67]	4.87 (< 0.001)	3.30	3 (0.35)	9	Fixed
Homozygous (TT vs. CC)	2.48 [1.72, 3.56]	4.90 (< 0.001)	4.30	3 (0.23)	30	Fixed
Heterozygous (CT vs. CC)	1.67 [1.26, 2.21]	3.35 (< 0.001)	5.24	3 (0.16)	43	Fixed
**−460C/T (rs833061) in Asian population from 3 case-control studies (766 cases and 799 controls)**						
Allelic (T-allele vs. C-allele)	1.58 [1.13, 2.22]	2.65 (0.008)	8.85	2 (0.01)	77	Random
Recessive (TT vs. CT + CC)	1.90 [1.03, 3.49]	2.06 (0.04)	12.40	2 (0.002)	84	Random
Dominant (CC vs. CT + TT)	0.49 [0.37, 0.65]	4.97 (< 0.001)	2.41	2 (0.30)	17	Fixed
Homozygous (TT vs. CC)	2.61 [1.78, 3.82]	4.93 (< 0.001)	3.62	2 (0.16)	45	Fixed
Heterozygous (CT vs. CC)	1.73 [1.29, 2.32]	3.69 (< 0.001)	4.21	2 (0.12)	53	Fixed

OR, odds ratio; 95% CI, 95% confidence interval.

A significant association was also observed in the subgroup of 766 Asian cases and 799 Asian controls in 3 studies [29–32] according to five genetic models (Table 4): allelic, OR 1.58, 95% CI 1.13–2.22, *P* = 0.008; recessive, OR 1.90, 95% CI 1.03–3.49, *P* = 0.04; dominant, OR 0.49, 95% CI 0.37–0.65, *P* < 0.001; homozygous, OR 2.61, 95% CI 1.78–3.82, *P* < 0.001; and heterozygous, OR 1.73, 95% CI 1.29–2.32, *P* < 0.001.

### Meta-analysis of studies on the −2578C/A (rs699947) polymorphism

**Figure 4:**
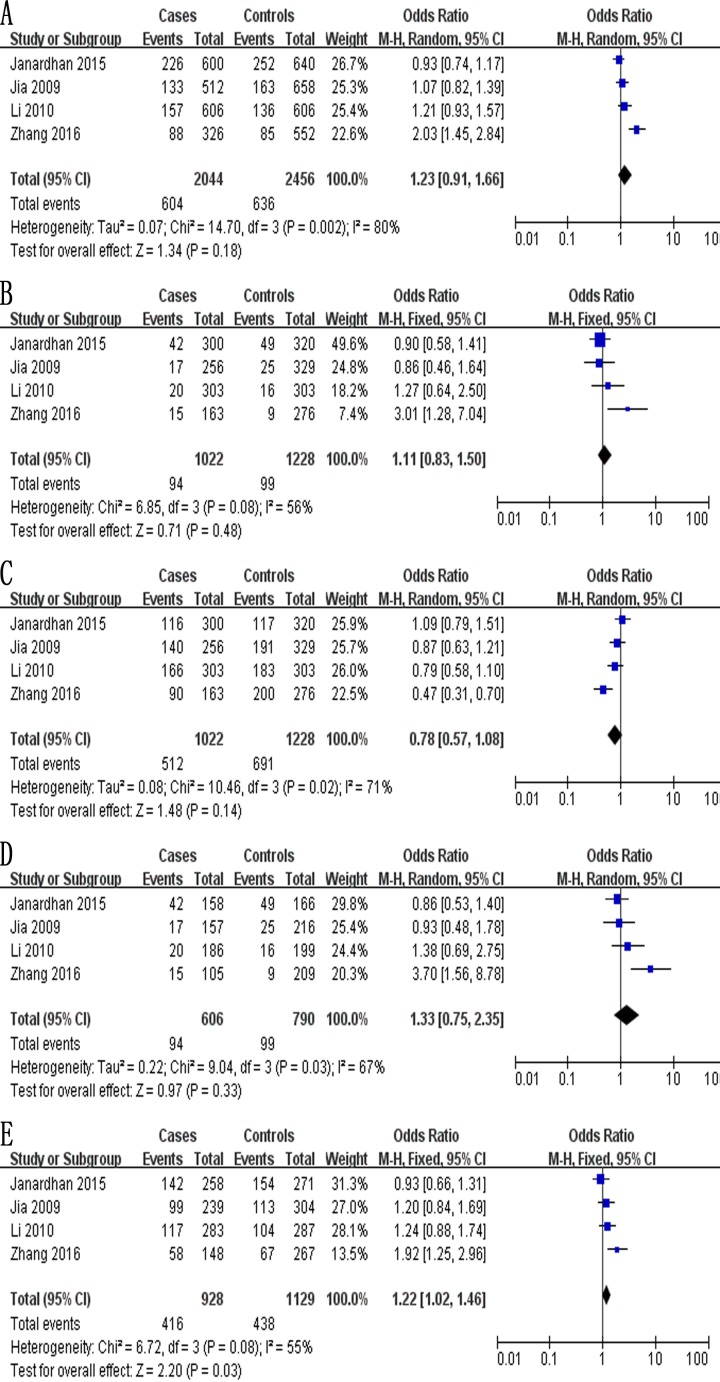
Forest plot describing the association between the −2578C/A polymorphism (rs699947) and risk of ovarian cancer across all study participants according to five genetic models (**A**) allelic (A-allele vs. C-allele), (**B**) recessive (AA vs. CA + CC), (**C**) dominant (CC vs. CA + AA), (**D**) homozygous (AA vs. CC) and (**E**) heterozygous (CA vs. CC).

The meta-analysis of a possible association between the −2578C/A polymorphism and ovarian cancer risk is summarized in Table 5. Based on the total study population (exclusively Asian) involving 1,022 cases and 1,228 controls, no evidence of an association was identified in four genetic models: allelic, OR 1.23, 95% CI 0.91–1.66, *P* = 0.18 (Figure 4A); recessive, OR 1.11, 95% CI 0.83–1.50, *P* = 0.48 (Figure 4B); dominant, OR 0.78, 95% CI 0.57–1.08, *P* = 0.14 (Figure 4C); and homozygous, OR 1.33, 95% CI 0.75–2.35, *P* = 0.33 (Figure 4D). In contrast, the CA genotype at −2578C/A was found to be a risk factor in the heterozygous model (OR 1.22, 95% CI 1.02–1.46, *P* = 0.03; Figure 4E).

**Table 5:** Overall meta-analysis of the association between the *−*2578C/A (rs699947) and risk of ovarian cancer

Genotype comparison and genetic model	OR [95 % CI]	Z (*P* value)	Heterogeneity of study design			Analysis model
			χ^2^	df (*P* value)	I^2^(%)	
**-2578C/A (rs699947) in total population from 4 case-control studies (1,022 cases and 1,228 controls)**						
Allelic (A-allele vs. C-allele)	1.23 [0.91, 1.66]	1.34 (0.18)	14.70	3 (0.002)	80	Random
Recessive (AA vs. CA + CC)	1.11 [0.83, 1.50]	0.71 (0.48)	6.85	3 (0.08)	56	Fixed
Dominant (CC vs. CA + AA)	0.78 [0.57, 1.08]	1.48 (0.14)	10.46	3 (0.02)	71	Random
Homozygous (AA vs. CC)	1.33 [0.75, 2.35]	0.97 (0.33)	9.04	3 (0.03)	67	Random
Heterozygous (CA vs. CC)	1.22 [1.02, 1.46]	2.20 (0.03)	6.72	3 (0.08)	55	Fixed

OR, odds ratio; 95% CI, 95% confidence interval.

### Meta-analysis of studies on the −1154G/A (rs1570360) polymorphism

**Figure 5:**
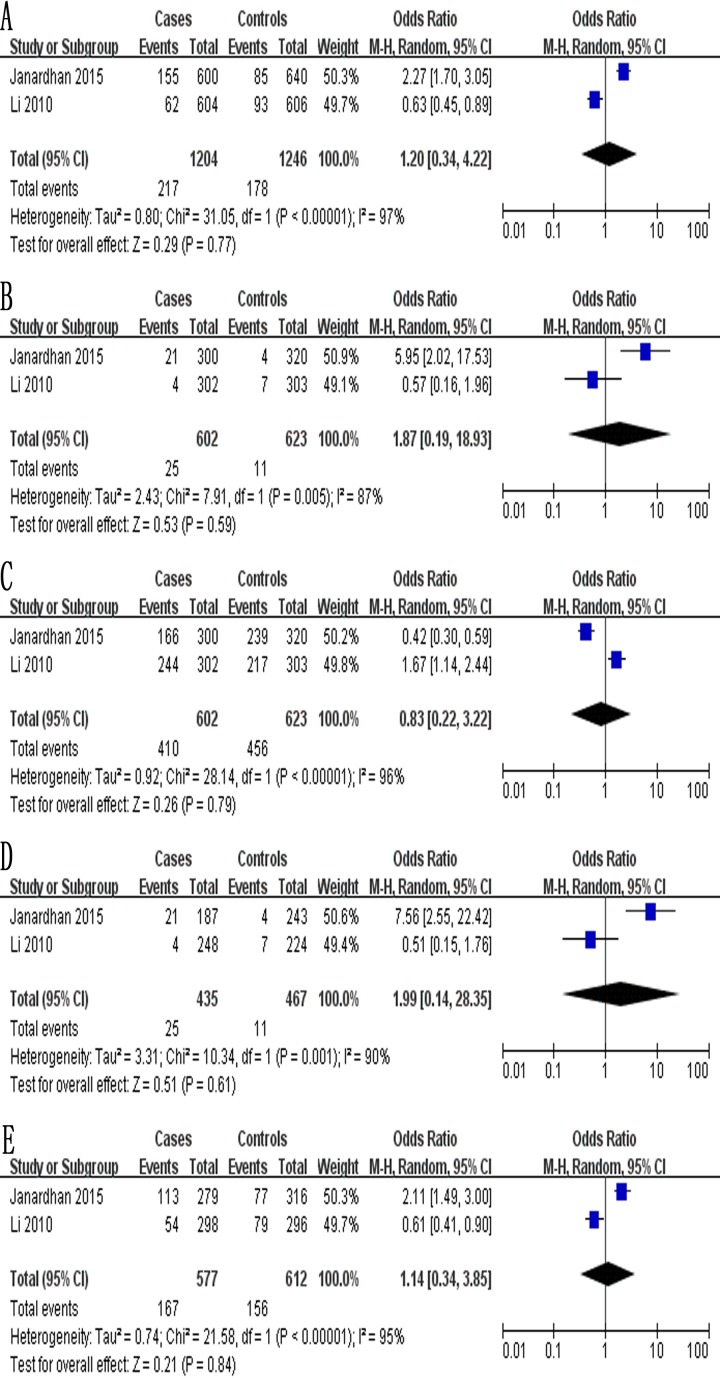
Forest plot describing the association between the −1154G/A polymorphism (rs1570360) and risk of ovarian cancer across all study participants according to five genetic models (**A**) allelic (A-allele vs. G-allele), (**B**) recessive (AA vs. GC + GG), (**C**) dominant (GG vs. GA + AA), (**D**) homozygous (AA vs. GG) and (**E**) heterozygous (GA vs. GG).

The meta-analysis of a possible association between the −1154G/A polymorphism and ovarian cancer risk is summarized in Table 6. Based on the total study population (exclusively Asian) involving 602 cases and 623 controls, none of the five genetic models indicated a significant association: allelic, OR 1.20, 95% CI 0.34–4.22, *P* = 0.77 (Figure 5A); recessive, OR 1.87, 95% CI 0.19–18.93, *P* = 0.59 (Figure 5B); dominant, OR 0.83, 95% CI 0.22–3.22, *P* = 0.79 (Figure 5C); homozygous, OR 1.99, 95% CI 0.14–28.35, *P* = 0.61 (Figure 5D); and heterozygous, OR 1.14, 95% CI 0.34–3.85, *P* = 0.84 (Figure 5E).

**Table 6:** Overall meta-analysis of the association between the −1154G/A (rs1570360) and risk of ovarian cancer

Genotype comparison and genetic model	OR [95 % CI]	Z (*P* value)	Heterogeneity of study design			Analysis model
			χ^2^	df (*P* value)	I^2^(%)	
**−1154G/A (rs1570360) in total population from 2 case-control studies (602 cases and 623 controls)**						
Allelic (A-allele vs. G-allele)	1.20 [0.34, 4.22]	0.29 (0.77)	31.05	1 (< 0.001)	97	Random
Recessive (AA vs. GC + GG)	1.87 [0.19, 18.93]	0.53 (0.59)	7.91	1 (0.005)	87	Random
Dominant (GG vs. GA + AA)	0.83 [0.22, 3.22]	0.26 (0.79)	28.14	1 (< 0.001)	96	Random
Homozygous (AA vs. GG)	1.99 [0.14, 28.35]	0.51 (0.61)	10.34	1 (0.001)	90	Random
Heterozygous (GA vs. GG)	1.14 [0.34, 3.85]	0.21 (0.84)	21.58	1 (< 0.001)	95	Random

OR, odds ratio; 95% CI, 95% confidence interval.

### Sensitivity analysis

The robustness of the meta-analysis of 4 studies [26, 29, 31, 32] examining a possible association between the −460C/T polymorphism and ovarian cancer risk was assessed by repeating the meta-analysis after excluding a study by Zhang et al. [32] in which the *P* value associated with HWE was less than 0.05. Deleting these data from the meta-analysis did not alter the results except in the recessive model, the results of which should therefore be interpreted with caution.

### Publication bias

**Figure 6:**
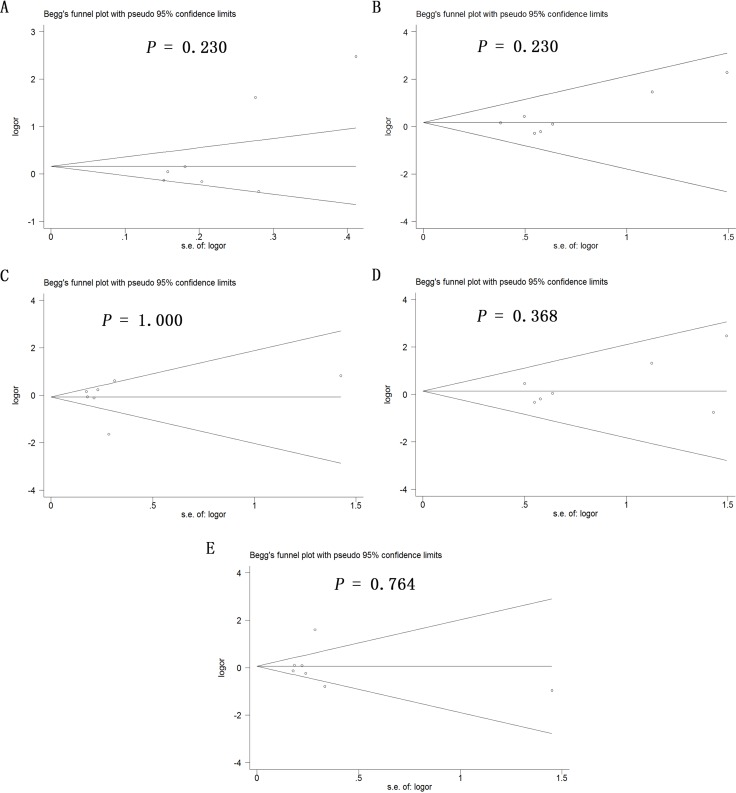
Begg's funnel plot to assess publication bias in the meta-analysis of a potential association between the +936C/T polymorphism (rs3025039) and risk of ovarian cancer across all study participants according to five genetic models (**A**) allelic (T-allele vs. C-allele), (**B**) recessive (TT vs. CT + CC), (**C**) dominant (CC vs. CT + TT), (**D**) homozygous (TT vs. CC) and (**E**) heterozygous (CT vs. CC).

**Figure 7:**
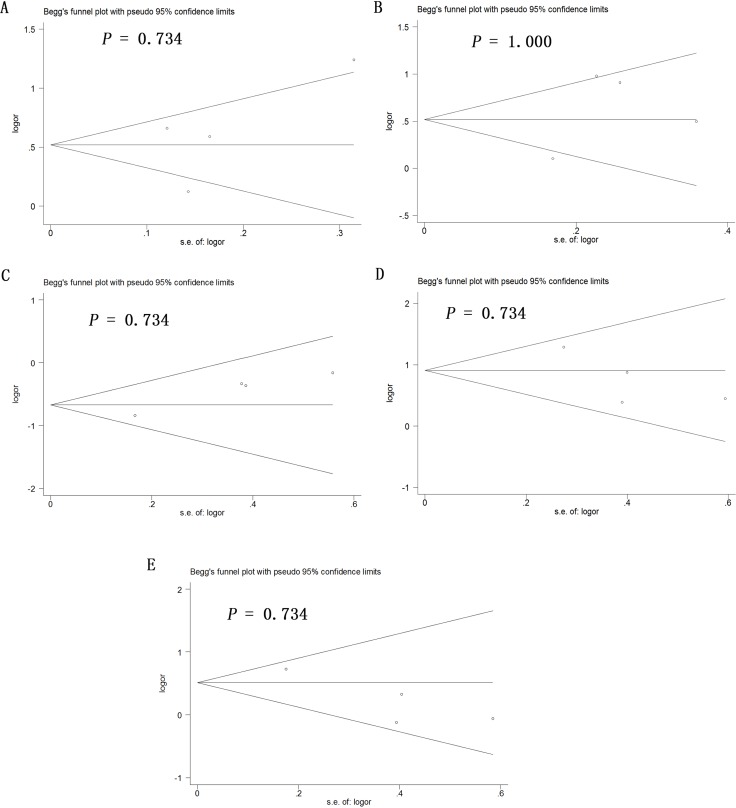
Begg's funnel plot to assess publication bias in the meta-analysis of a potential association between the −460C/T polymorphism (rs833061) and risk of ovarian cancer across all study participants according to five genetic models (**A**) allelic (T-allele vs. C-allele), (**B**) recessive (TT vs. CT + CC), (**C**) dominant (CC vs. CT + TT), (**D**) homozygous (TT vs. CC) and (**E**) heterozygous (CT vs. CC).

**Figure 8:**
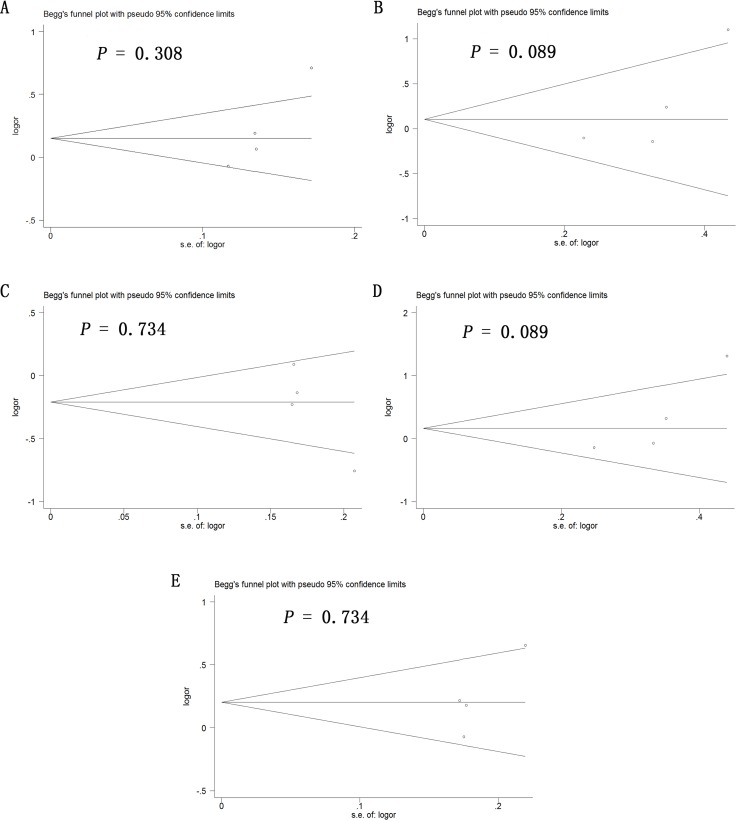
Begg's funnel plot to assess publication bias in the meta-analysis of a potential association between the −2578C/A polymorphism (rs699947) and risk of ovarian cancer across all study participants according to five genetic models (**A**) allelic (A-allele vs. C-allele), (**B**) recessive (AA vs. CA + CC), (**C**) dominant (CC vs. CA + AA), (**D**) homozygous (AA vs. CC) and (**E**) heterozygous (CA vs. CC).

Potential publication bias in this meta-analysis was assessed using Begg's funnel plot. No obvious asymmetry was observed in Begg's funnel plots of allelic modeling of the polymorphisms +936C/T (Figure 6), −460C/T (Figure 7) or −2578C/A (Figure 8). *P* values for Begg's test were greater than 0.05 for the +936C/T results based on all the genetic models: allelic, *P* = 0.230; recessive, *P* = 0.230; dominant, *P* = 1.000; homozygous, *P* = 0.368; and heterozygous, *P* = 0.764. Similarly, *P* values were greater than 0.05 for the −460C/T results (allelic, *P* = 0.734; recessive, *P* = 1.000; dominant, *P* = 0.734; homozygous, *P* = 0.734; heterozygous, *P* = 0.734) and for the −2578C/A results (allelic, *P* = 0.308; recessive, *P* = 0.089; dominant, *P* = 0.734; homozygous, *P* = 0.089; heterozygous, *P* = 0.734). These results suggest no potential publication bias in the included data on +936C/T, −460C/T and −2578C/A polymorphisms. Begg's test was not applied to data on the −1154G/A polymorphism because of the small number of publications.

## DISCUSSION

The number of case-control studies exploring the influence of VEGF-A polymorphisms on ovarian cancer risk has grown in recent years [26–32]. Limited sample size and ethnic differences among the various populations examined have contributed to a lack of consensus in this literature, so we conducted this comprehensive meta-analysis to evaluate the association of ovarian cancer risk with four polymorphisms in the VEGF-A gene (+936C/T, −460C/T, −2578C/A and −1154G/A). Our meta-analysis suggests that the −460C/T polymorphism is significantly associated with ovarian cancer risk across the total population as well as the Asian population. In contrast, none of the five genetic models suggested a significant association between the + 936C/T polymorphism and ovarian cancer risk in Asian populations in general or in Chinese populations specifically. None of the five genetic models suggested a significant association between the −1154G/A polymorphism and ovarian cancer risk across the entire study population.

While the present meta-analysis was being conducted, Zhang et al. [33] published a meta-analysis of the relationship between ovarian cancer risk and the three polymorphisms +936C/T, −460C/T, and −2578C/A. Similar to their results, we found that the CT genotype at +936C/T may act as a protective factor in Caucasian populations. On the other hand, our meta-analysis contrasts with the previous one because we found the −460C/T polymorphism to be significantly associated with ovarian cancer risk across the total population as well as the Asian subpopulation, and the CA genotype at −2578C/A to be associated with cancer risk across the total population, whereas that previous meta-analysis did not report either association. This discrepancy may reflect the fact that we included two large case-control studies involving all four VEGF-A polymorphisms absent from the previous meta-analysis, leading to much larger sample sizes for meta-analysis of 2578C/A and −460C/T polymorphisms in our work. In addition, we meta-analyzed the relationship between −1154G/A polymorphism and ovarian risk, which was not examined in that previous meta-analysis. Therefore, our meta-analysis provides new evidence for the important role of VEGF-A polymorphisms in ovarian cancer development. To the best of our knowledge, the present study is the most comprehensive and robust meta-analysis of these genetic polymorphisms and ovarian cancer.

Despite the potential insights it offers, the present study has several limitations that may affect interpretation of the results. First, the *P* value for HWE was less than 0.05 in the case-control study by Zhang et al. [32] on the −460C/T polymorphism. These results suggest that this study population may not be representative of the broader target population. Nevertheless, sensitivity analyses showed that deleting these data from the meta-analysis did not alter the results except in the recessive model, which is unlikely to significantly affect the observed significant relationship between −460C/T polymorphism and ovarian cancer risk. Second, our exclusion of unpublished data and of papers published in languages other than English and Chinese may have biased our results. Third, the studies may be subject to performance bias, attrition bias and reporting bias, although Newcastle–Ottawa scores were at least 6 for all 7 studies, indicating high quality. Lastly, the results may be affected by additional confounding factors, such as age, obesity, type of cancer, or other factors, and we could not take this into account in the meta-analyses because studies either did not report these baseline data or they aggregated the data in different ways. Thus, these conclusions should be verified in large, well-designed studies.

In conclusion, this meta-analysis indicates that there may be a significant association between the −460C/T polymorphism and ovarian cancer risk. The CA genotype at −2578C/A may be a risk factor in the total population, while the CT genotype at +936C/T may be a protective factor in the Caucasian population. The −1154G/A polymorphism may not be related to ovarian cancer risk.

## MATERIALS AND METHODS

### Search strategy

PubMed, EMBASE, Google Scholar and the Chinese National Knowledge Infrastructure databases were systematically searched up to April 12, 2017 for clinical and experimental case-control studies published in English or Chinese that assessed potential associations of ovarian cancer risk with at least one of the following polymorphisms in the VEGF-A gene: +936C/T (rs3025039), −460C/T (rs833061), −2578C/A (rs699947), and −1154G/A (rs1570360). The following search strings were used: *vascular endothelial growth factor +936C/T, vascular endothelial growth factor* −*460C/T, vascular endothelial growth factor* −*2578C/A, vascular endothelial growth factor* −*1154G/A, rs3025039, rs833061, rs699947,* and *rs1570360.* Searches were also conducted with each of these eight terms AND each of the following terms: *polymorphism, polymorphisms, SNP, variant, variants, variation, genotype, genetic* or *mutation.* Lastly, searches were conducted with each of the above terms AND each of the following: *ovarian cancer, ovarian carcinoma or OC*. Reference lists in identified articles and reviews were also searched manually to identify additional eligible studies.

This literature and meta-analysis were performed in accordance with the guidelines and recommendations of the Preferred Reporting Items for Systematic Reviews and Meta-Analyses (Supplementary Table 1) [34].

### Inclusion criteria

To be included in our review and meta-analysis, studies had to satisfy the following criteria: (1) a case-control design was used to assess the association of at least one of the four target polymorphisms with ovarian cancer risk in humans; (2) full text was available, and sufficient data were reported to estimate an odds ratio (OR) with 95% confidence interval (CI); and (3) genotype frequencies were reported. If multiple publications from the same research group appeared to report data for the same cases and controls, we included only the most recent publication in our meta-analysis.

### Data extraction

Two authors (CHX and ZHH) independently extracted the following data from included studies: first author's family name, year of publication, ethnicity, country of origin, testing methods, type of controls, *P* value for HWE in controls, numbers and genotypes of cases and controls, frequencies of genotypes in cases and controls. Discrepancies were resolved by consensus.

### Assessment of methodological quality

The quality of the included studies was assessed independently by two authors (CHX and ZHH) according to the Newcastle–Ottawa Scale [35]. This scale awards a maximum of 9 points to a study, with higher scores indicating better quality. Differences in quality score outcomes between the two assessors were solved by consensus. If consensus was not reached, a third assessor (HX) was consulted for the final decision.

### Statistical analysis

Unadjusted odds ratios (ORs) with 95% confidence intervals (95% CI) were used to assess the strength of the association of each of the four target polymorphisms with ovarian cancer risk, based on genotype frequencies in cases and controls. The significance of pooled ORs was determined using the *Z* test, with *P* < 0.05 defined as the significance threshold. Meta-analysis was conducted using a fixed-effect model when *P* > 0.10 for the *Q* test, indicating lack of heterogeneity among studies; otherwise, a random-effect model was used. All statistical tests for meta-analyses were performed using Review Manager 5.2 (Cochrane Collaboration). Publication bias was assessed using Begg's funnel plot Stata 14.0 (Stata Corp, College Station, TX, USA), with *P* < 0.05 considered statistically significant.

## SUPPLEMENTARY MATERIALS TABLE




