# Supplementary material for: Association of four genetic polymorphisms in the vascular endothelial growth factor-A gene and development of ovarian cancer: a meta-analysis
Source: Oncotarget. 2017 Aug 21;8(42):73063–78. doi: 10.18632/oncotarget.20379 (PMC5641192; doi:10.18632/oncotarget.20379)
Supplement: Supplementary file 1 [file oncotarget-08-73063-s001.pdf]

Association of four genetic polymorphisms in the vascular endothelial growth factor-A gene and development of ovarian cancer: a meta-analysis

SUPPLEMENTARY MATERIALS

Supplementary Table 1: PRISMA 2009 checklist. See Supplementary_Table_1
